# Bridging communication gaps across the biomedical ecosystem

**DOI:** 10.3389/fsysb.2026.1939110

**Published:** 2026-10-12

**Authors:** Elizabeth A. Burke, Samantha N. Schumm, Amy Jobe, Kara Whelply, Ishita Das

**Affiliations:** The Milken Institute, Washington, DC, United States

**Keywords:** public engagement, science communication, scientific research, social media, public trust

## Abstract

The current landscape of science communication is undergoing rapid transformation, shaped by profound societal shifts including the COVID-19 pandemic, the rise of social media, the decline of traditional news media, and the growing influence of large language models. These changes have fundamentally altered how the biomedical community and the public share, consume, and trust scientific information. This article contextualizes these changes, explains why redesigning the science communication ecosystem is urgent, and offers recommendations to improve accuracy, strengthen trust, and deepen public engagement. Specifically, philanthropy can play a key role by driving momentum and optimizing the flow of scientific information across three stages: (1) scientific discovery to publication, (2) interpretation and distribution, and (3) public engagement.

## Introduction

Over the past decade, the ways in which scientific information is produced, shared, and consumed have dramatically shifted. With communications during the COVID-19 pandemic serving as an example, it is clear that social media’s rise, the decline of traditional news platforms, and the emergence of AI platforms as knowledge intermediaries have reshaped how science reaches the public. While information is more accessible than ever, audiences have also become increasingly divided along political and ideological lines, accelerating the spread of misinformation and making it more difficult to assess the credibility of sources (Milkoreit and Smith, 2024). As a result, long-standing assumptions about how scientific knowledge flows from the lab to the public and how trust is built are changing.

This moment presents both challenges and opportunities. Scientific advances are increasingly central to addressing global challenges including climate change, public health, and sustainable development; however, the institutions and incentives supporting science communication have not evolved at the same pace, limiting the effective translation of scientific knowledge to policymakers and the public (Huang et al., 2024; Kroon and Eickholt, 2024). Like the childhood game of “telephone,” where messages become distorted as they are passed along, breakdowns across the science communication pipeline can misrepresent evidence, obscure uncertainty, or limit accessibility, weakening public understanding and trust. This article examines the current state of science communication and proposes practical solutions to reduce these breakdowns and strengthen direct engagement between scientists and the public, while highlighting philanthropy’s role in catalyzing these solutions. This evolving landscape has created a new role for philanthropy to engage by supporting trusted intermediaries, fostering innovation in science communication, and helping build stronger connections between scientists and society.

Figure 1 presents a conceptual model tracing how scientific information moves from discovery to public engagement. It highlights the interconnected roles of researchers, journals, media, policymakers, institutions, and the public, while identifying key points where communication can falter, including misinterpretation, inaccessible language, silos, or media amplification. This article follows this flow across three stages: (1) scientific discovery to publication, (2) interpretation and distribution, and (3) public engagement. Together, these stages highlight strengths, gaps, and opportunities that lay the groundwork for a more transparent, inclusive, and resilient science communication ecosystem.

**FIGURE 1:**
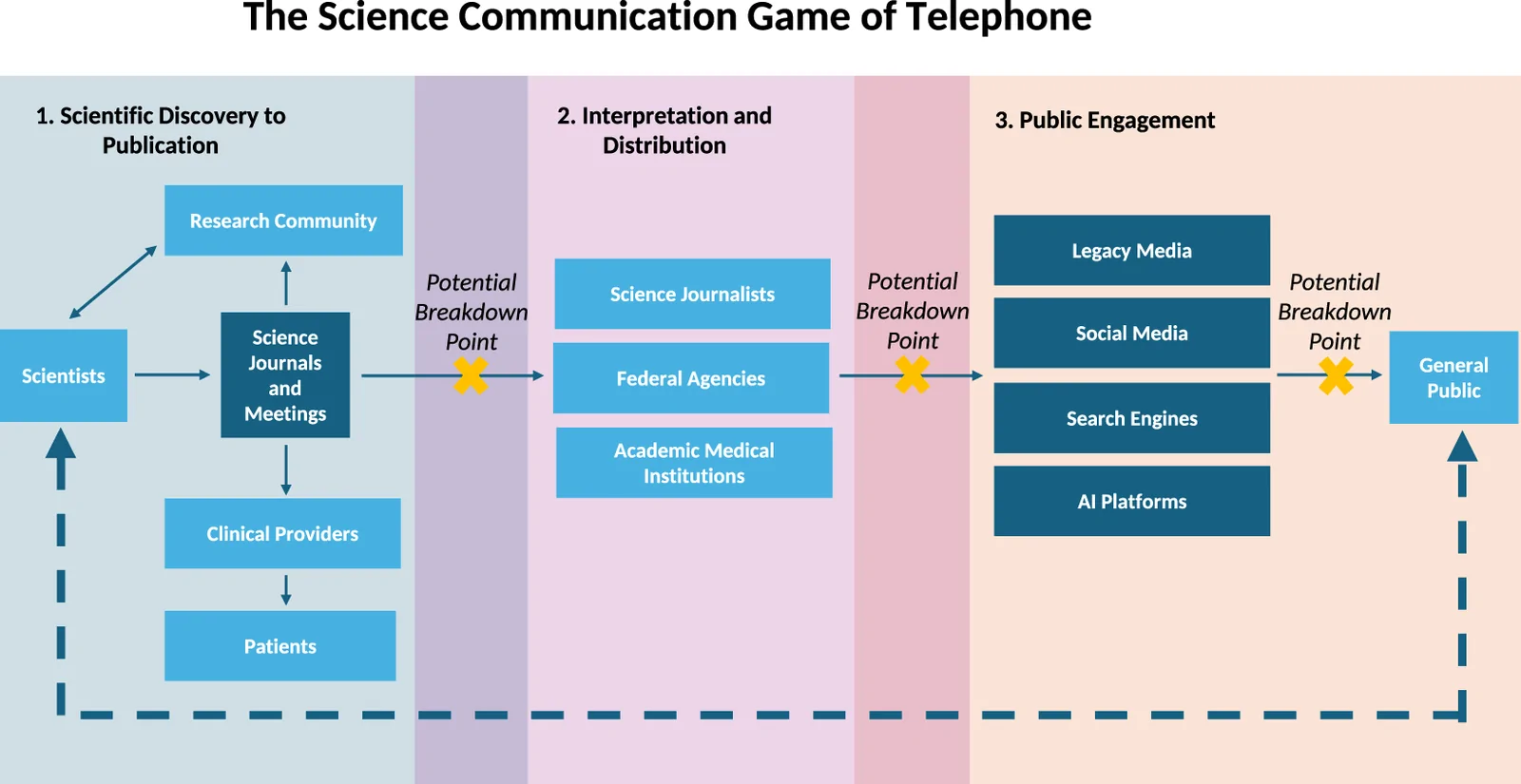
The Science Communication Game of Telephone. Potential breakdown points across the flow of information from scientific discovery all the way to public engagement are highlighted This article discusses vulnerabilities at these breakdown points and offers recommendations to address them, with the goal of promoting effective communication between the scientific community and the public.

## Stage 1: scientific discovery to publication

Once scientists and clinicians draw conclusions from their research, they communicate their findings to the broader biomedical community through peer-reviewed journal articles and presentations at scientific and medical conferences. This exchange of knowledge is critical for informing future research and clinical care, as well as fostering collaboration among researchers.

### Barriers to publishing and accessing scientific information

Traditional peer-reviewed academic journals remain the trusted gold standard for communication within the biomedical community because of their rigorous scientific and editorial oversight. However, accessibility challenges can hinder authors from sharing findings on their own terms and can limit dissemination across the broader biomedical community.

For authors, several factors can complicate, delay, or prevent publication. These include potentially prohibitive manuscript submission fees, a high likelihood of rejection, lengthy peer-review and editorial processes (Drozdz and Ladomery, 2024), and publishing practices that prioritize positive findings and novel discoveries over building a more complete evidence base that includes confirmatory studies and negative data. Among these factors, slow peer review is a substantial contributor to publication delays, due to the poor ratio of available peer reviewers to the number of manuscripts in need of review has worsened since the COVID-19 pandemic (Drozdz and Ladomery, 2024). That trend has been compounded as the number of manuscript submissions has increased, due in part to LLM usage to draft manuscripts (Messeri and Crockett, 2026).

For potential readers, full-length journal articles are often inaccessible behind expensive paywalls. Published work may also be difficult for many biomedical professionals outside a given specialty to understand. Field-specific terminology and dense writing that prioritizes technical precision over clear storytelling can limit accessibility, an understandable yet unfortunate result of today’s hypercompetitive publishing and funding environments.

To tackle some of these challenges, many journals have introduced policies to make information more accessible, including open access options and requiring authors to provide plain-language summaries or graphical abstracts. However, adoption of these policies is inconsistent, as many remain unclear or unenforced. For example, plain-language summaries remain relatively uncommon or technical (The Publication Plan, 2025; Baróniková et al., 2024), while graphical abstracts may create accessibility barriers for some audiences.

### Recommendations to improve communication within the biomedical community

Improving publication quality and accessibility requires effort and commitment from multiple parties across the research ecosystem. Academic journals can leverage their data on readership, citation metrics, digital engagement, and more to evaluate which editorial policies are most effective and inform their guidelines for publications, data sharing, and plain-language summaries (Pal et al., 2024). Research institutions can improve the publications of their biomedical researchers and clinicians by retaining department- or school-level support from in-house statisticians, graphic designers, technical writers, and communications specialists. By providing all research teams with equitable access to these staff, institutions can improve the overall quality and accessibility of publications, regardless of a research team’s size, financial resources, or collective experience.

Biomedical researchers and clinicians can take several steps to improve the accessibility of their work. First, authors should tailor their writing to the expertise and professional backgrounds of their intended audiences. For instance, a publication written for biomedical engineers, orthopedic surgeons, and health policy experts should avoid discipline-specific jargon that might not translate broadly. Second, context is as important as content: authors should clearly describe the conditions under which their findings apply while acknowledging limitations, uncertainty, and sources of subjectivity (Roney et al., 2026). Third, visual communication, including figures, graphical abstracts, conference posters, and presentations, can either enhance or hinder understanding. Developing fluency in creating accessible, compelling, and accurate graphics (Tufte, 2018) and presentations (Center for Teaching and Learning, Stanford University, 2013) is paramount. Finally, authors can publish their work in preprint outlets to facilitate the timely dissemination of critical findings, especially when publication costs are prohibitive.

Philanthropic funders have the agency to incentivize best practices in communication among funding recipients. For example, through a partnership with the authors’ institution (the Milken Institute’s Science Philanthropy Accelerator for Research and Collaboration), the Misophonia Research Fund engaged a communications firm to strengthen their grantees’ communication skills and hosted a virtual forum^1^ where they shared findings with the biomedical community and the public (soQuiet, 2023). Philanthropic funders can also encourage or require recipient institutions to retain publication support staff and promote collaborative, open science practices.

## Stage 2: interpretation and distribution

Once scientific research is published in academic journals, findings are picked up, translated for various purposes, and distributed to different audiences, including the general public. This process of translating and relaying scientific findings is especially susceptible to breakdowns in communication because it is easy for intermediaries to introduce inaccuracies and misinterpretations as information is consumed, analyzed, interpreted, and repackaged for a variety of audiences. While many actors play intermediary roles, including academic medical institutions and federal agencies, science journalism is a critical and increasingly vulnerable part of relaying scientific information.

### The role and challenges of science journalism

Central to this relay system is science journalism, which plays a key role in how society learns about and discusses science and its impact. However, science journalism faces significant challenges in a changing media environment. As the advertising-based model that historically supported news platforms becomes outdated, legacy media organizations are experiencing substantial disruption, resulting in reduced reporting capacity, especially in specialized areas such as science journalism.

With the US losing an average of over two newspapers per week (Metzger, 2025), the spread of “news deserts” with few to no local news sources reduces media capacity and the potential for information to permeate many communities. Local news is highly trusted and has significant reach within its respective communities, making it a key part of the information ecosystem and a vehicle for disseminating accurate scientific information (National Academies of Sciences Engineering, and Medicine et al., 2025). The contraction of traditional news media also means fewer opportunities for formal specialized training in science journalism. In fact, most journalists are generalists; fewer than 3% bring an undergraduate science major to their reporting (Menezes, 2018; Sachsman et al., 2008). These shifting media dynamics are altering how scientific information reaches the public.

Increasingly, clinical and research scientists employed at universities and academic medical institutions participate in the news ecosystem by posting on social media, authoring articles for public audiences, and engaging with reporters. While these opportunities facilitate more direct communication between researchers and the public, scientists are not typically trained in public communication. Without that training, they risk misrepresenting nuance or uncertainty, inadvertently undermining public trust in science. Prioritizing proactive, transparent, and lay-level communication can help combat this risk and ensure that accurate information spreads.

### Risk of misinformation

As journalism struggles, the risk of spreading inaccurate scientific information increases, especially as social media has become a dominant means of information-sharing. Social media can disseminate information quickly and widely, but misinformation can proliferate rapidly and thrive in the absence of easily accessible, credible information. Blame for misinformation often falls on social media, but misinformation stems from a variety of sources, including authoritative and sometimes even reputable ones—for example, corporations, government offices, entertainment media, press offices, individual scientists, healthcare professionals, or research institutions (National Academies of Sciences, Engineering, and Medicine, 2025). AI platforms and LLM chatbots now also serve as distribution channels, with the potential to amplify both accurate and misleading scientific information. While misinformation may arise unintentionally, it is imperative for everyone who participates in this ecosystem to share accurate, reliable scientific information to buttress public trust in science.

### Recommendations to improve scientific reporting

Strategies to strengthen the dissemination of high-fidelity scientific reporting include expanding training programs and fellowships for science journalists, fostering collaborations between scientists and media professionals, and increasing philanthropic investment in journalism and local news ecosystems.

Efforts to grow expertise in science journalism include training programs and fellowships^2^ that support specialized training, especially for early-career journalists, in areas such as climate and health. In one notable example, the Knight Science Journalism^3^ Academic-Year fellows spend a year at MIT exploring science and technology research projects and honing technical skills to bring back into the field.

Scientific societies can educate and train scientists to work with journalists and communicate with the public. Developed by the American Association for the Advancement of Science, SciLine^4^ connects journalists and scientists. The service offers journalists free training sessions, resources to strengthen reporting with scientific evidence, and a free matching service that pairs reporters with scientific experts for news stories. Scientists can register to be part of SciLine’s network and access media training and advice. Best practices emphasize presenting scientific findings as part of a process that involves inquiry and uncertainty to show how scientific knowledge evolves (Ophir and Jamieson, 2021).

Universities and academic medical centers also have a clear and important role in communication training. As just one example of how universities are incorporating communication skills alongside scientific training, Stony Brook University, in collaboration with the Alan Alda Center for Communicating Science^5^ offers graduate students the opportunity to earn a certification in science communication during their doctoral training. Furthermore, many university press offices offer valuable communication support to researchers and should ensure that scientists are aware of those resources and how to access them.

Philanthropy has a real opportunity to drive progress in building capacity and expertise in reporting, especially in science journalism. A philanthropic effort to revitalize journalism is Press Forward^6^—a national network that aims to strengthen local news and close coverage gaps. Led by the MacArthur Foundation, the initiative is bringing new funders into journalism by reducing barriers to entry—for instance, providing technical assistance to other funders and making it simple to contribute to collective funds (Scutari, 2025). Given that much philanthropic giving is place-based and grounded in personal interests or experiences, science journalism can appeal to many funders because it operates at the intersection of those domains.

With diminished capacity in journalism, the process of translating scientific findings is under greater stress, giving misinformation more opportunity to take hold. Philanthropy can play a key role in bolstering this information ecosystem by supporting training for both journalists and scientists to improve communication around complex, technical topics.

## Stage 3: public engagement

Scientific and health information reaches the public through a range of intermediaries and an expanding array of channels, including healthcare providers, news outlets, social media influencers, search engines, AI platforms, and informal social networks. Engagement with this information is often situational: individuals actively seek scientific guidance when faced with personal health concerns, but at other times their exposure is largely passive, shaped by headlines, algorithms, and social conversations. In this increasingly crowded information landscape, the public is not only exposed to more information than ever before but must also actively decide which sources to trust.

### Public polls point to a way forward

In April 2026, the Pew Research Center reported that 85% of Americans obtain science and health information at least sometimes from healthcare providers, 66% from people with similar health experiences, and 60% from major health websites such as WebMD or Mayo Clinic (Pasquini et al., 2026). Less frequently cited sources included journalists and news organizations (46%), government health agencies (45%), social media (36%), and AI chatbots (22%). While these diverse channels have broadened access to information, they have not necessarily increased confidence in using it to improve health outcomes. In fact, global and US public confidence in the ability to find reliable answers and make informed health decisions has significantly declined in the past year (Edelman, 2026). A key reason for this paradox is the multiple layers of reinterpretation that occur in the current science communication pipeline, as shown in Figure 1. As a result, different platforms often deliver conflicting or oversimplified narratives, leaving audiences uncertain about what to believe and more likely to gravitate toward the most prominent or emotionally compelling voices. Thus, the same forces that have democratized access to scientific knowledge have simultaneously made it more difficult to evaluate and trust.

The COVID-19 pandemic vividly exposed the consequences of this fragmented communication system. Rapidly evolving evidence required frequent updates to public health guidance, but inconsistent messaging and insufficient explanation of scientific uncertainty often led to confusion. At the same time, social media platforms amplified both accurate and misleading information at unprecedented speed. In such an environment, simplified or sensational claims frequently outpaced nuanced, evidence-based explanations, contributing to widespread misinformation and eroding trust in public health institutions.

Understanding what people value in information sources provides a path toward addressing these challenges. Approximately 75% of Americans prioritize sources that demonstrate relevant medical expertise, disclose conflicts of interest, and present information clearly and accessibly. However, preferences vary across populations. Individuals with lower incomes or less formal education are more likely to prioritize likability and relatability, while those without health insurance often emphasize convenience, including access through social media and AI platforms (Pasquini et al., 2026). At the same time, surveys indicate that roughly 70% of Americans report at least moderate trust in scientists to act in the public’s best interest (Annenberg Public Policy Center, 2026). Taken together, these findings suggest that trust in medical and scientific expertise remains relatively strong, but access to expert-driven information is often limited by barriers of clarity, convenience, and reach.

### Recommendations to improve public access, engagement, and trust

This gap between trusted sources and accessible communication represents a central weakness in the current science communication ecosystem. Information directly from scientists and healthcare providers is often perceived as authoritative but can be dense, difficult to access, or insufficiently tailored to public audiences. As a result, individuals may turn to more accessible but less reliable intermediaries.

Addressing this challenge will require coordinated, system-level action. Academic medical centers and hospitals, which employ a large proportion of the nation’s physicians and scientists, are uniquely positioned to improve public-facing communication. By incentivizing faculty, trainees, and clinicians to engage in outreach—both in person and digitally, including on social media—and by pairing them with professional science communicators, these institutions can expand the reach and clarity of expert information. The Mayo Clinic and its Academic Appointments and Promotions Committee have included digital and social media scholarship among the criteria considered for advancement, providing an incentive for its investigators to prioritize public engagement (Cabrera et al., 2017). At the community level, partnerships with local organizations can further enhance trust and accessibility. Training trusted community members to communicate evidence-based information can help bridge cultural and social gaps that formal institutions may struggle to address. For example, the Barbershop Talk with Brothers program, a partnership between the Arthur Ashe Institute for Urban Health and State University of New York Downstate Medical Center, provides health advocacy training to barbers who are trusted advisors in the Brooklyn community (Chau et al., 2023). Efforts like this, however, require sustained collaboration and mutual investment between scientific institutions and community-based organizations.

Finally, broader structural incentives are needed to make public communication a core component of scientific and medical work. Governments and funding agencies can support this shift by tying grants, accreditation, or research funding to demonstrated public communication. Philanthropic and nonprofit organizations can further accelerate progress by funding programs such as the Association for Research in Vision and Ophthalmology (ARVO) Science Communication Training Fellowship^7^ that integrate science communication training and outreach into academic and clinical careers.

In summary, the challenge is not a lack of public trust in science itself, but a misalignment between trust, accessibility, and communication pathways. Bridging this gap requires strengthening the direct connection between experts and the public, as shown by the dotted arrow in Figure 1—ensuring that accurate, transparent, and understandable information is not only available, but easy to find, engage with, and trust.

## Conclusion

The science communication ecosystem is at a pivotal moment. As access to information has expanded and new intermediaries have reshaped how science is produced, shared, and consumed, the pathways connecting discovery to public understanding have grown more complex and fragile. As described above, breakdowns can occur at every stage, fragmenting audiences, distorting evidence, and making it harder for the public to assess credibility. Moving beyond this modern “telephone game,” where information is easily altered as it travels, requires a coordinated approach.

Building a more resilient system will depend on strengthening connections across the pipeline and rethinking how scientific knowledge flows in a decentralized information environment. This includes aligning incentives, equipping scientists to engage more directly with diverse audiences, and embedding accessibility and transparency throughout the research and communication process. As this ecosystem evolves, philanthropy is uniquely positioned to serve as a catalytic partner, providing risk-tolerant capital to test new models of science communication, strengthen independent science journalism, support cross-sector collaboration, and build the infrastructure needed to connect research with public understanding. Rebuilding and maintaining trust in science will require not only producing high-quality knowledge, but ensuring that knowledge moves clearly, credibly, and reliably through modern information channels and reaches people in ways that are accessible, understandable, and meaningful.

## Data Availability

The original contributions presented in the study are included in the article/supplementary material, further inquiries can be directed to the corresponding author.

## Footnotes

1
https://www.soquiet.org/care2023

2
https://healthjournalism.org/fellowships

3
https://ksj.mit.edu/

4
https://www.sciline.org/

5
https://www.aldacenter.org/

6
https://www.pressforward.news/

7
https://www.arvo.org/professional-development/science-communication-training-fellowship

## Author’s note

The Milken Institute Science Philanthropy Accelerator for Research and Collaboration (SPARC) works with philanthropists and nonprofit organizations to create high-impact initiatives that accelerate biomedical research and turn innovation into real-world health improvements. Through our work, we actively work to build a bridge between the bench and the bedside by working with researchers, patient advocates, and funders.

## References

[B2] Annenberg Public Policy Center (2026). Scientists esteemed by public, with vaccine scientists seen as similar to scientists in general. Available online at: https://www.annenbergpublicpolicycenter.org/scientists-esteemed-by-public-with-vaccine-scientists-seen-as-similar-to-scientists-in-general/ (Accessed June 15, 2026).

[B5] Baróniková S. Rosenberg A. Winchester C. C. Philippon V. Gordon J. Osório J. (2024). Publisher perspectives on plain language summaries of scientific publications: an open pharma survey. Med. Writ. 33, 54–61. 10.56012/pzwh6593

[B6] Cabrera D. Vartabedian B. S. Spinner R. J. Jordan B. L. Aase L. A. Timimi F. K. (2017). More than likes and tweets: creating social media portfolios for academic promotion and tenure. J. Grad. Med. Educ. 9 (4), 421–425. 10.4300/JGME-D-17-00171.1 28824752 PMC5559234

[B7] Center for Teaching and Learning, Stanford University (2013). Creating effective slides: design, construction, and use in science. Available online at: https://www.youtube.com/ (Accessed June 15, 2026).

[B8] Chau M. M. Ahmed N. Pillai S. Telzak R. Fraser M. Islam N. S. (2023). Community-based organizations as trusted messengers in health. Hastings Cent. Rep. 53 (Suppl. 2), S91–S98. 10.1002/hast.1529 37963042 PMC10939007

[B9] Drozdz J. A. Ladomery M. R. (2024). The peer review process: past, present, and future. Br. J. Biomed. Sci. 81, 12054. 10.3389/bjbs.2024.12054 38952614 PMC11215012

[B10] Edelman (2026). 2026 edelman trust barometer special report: trust and health. Available online at: https://www.edelman.com/trust/2026/trust-barometer/special-report-health (Accessed June 15, 2026).

[B11] Huang L. Swain R. B. Jeppesen E. Wang H. Zeng J. Guo H. (2024). Harnessing science, technology, and innovation to drive synergy between climate goals and the SDGs. Innov. (Camb) 5, 100693. 10.1016/j.xinn.2024.100693 39399226 PMC11467660

[B13] Kroon C. Eickholt B. J. (2024). Great science does not communicate itself: but who should and how? Brain 147 (9), 2911–2912. 10.1093/brain/awae217 38961711

[B14] Menezes S. (2018). Science training for journalists: an essential tool in the post-specialist era of journalism. Front. Commun. 3, 4. 10.3389/fcomm.2018.00004

[B28] Messeri L. Crockett M. J. (2026). The uncritical adoption of AI in science is alarming ‐ we urgently need guard rails. Nat. 653 (8115), 675–676. 10.1038/d41586-026-01557-x 42156510

[B15] Metzger Z. (2025). The State of Local News. Evanston, Illinois: Northwestern University. Available online at: https://localnewsinitiative.northwestern.edu/projects/state-of-local-news/2025/report/ (Accessed July 14, 2026).

[B16] Milkoreit M. Smith E. K. (2024). Rapidly diverging public trust in science in the United States. Public Underst. Sci. 34, 616–627. 10.1177/09636625241302970 39644137 PMC12177194

[B17] National Academies of Sciences Engineering, and Medicine (2025). Understanding and Addressing Misinformation About Science. Editors Viswanath K. Taylor. T. E. Rhodes H. G. (Washington, D.C.: National Academies Press). 10.17226/27894

[B18] Ophir Y. Jamieson K. H. (2021). The effects of media narratives about failures and discoveries in science on beliefs about and support for science. Public Underst. Sci. 30, 1008–1023. 10.1177/09636625211012630 34000907

[B19] Pal A. Arnet I. Elger B. S. Wangmo T. (2024). Practices and barriers in developing and disseminating plain-language resources reporting medical research information: a scoping review. Patient 17, 493–518. 10.1007/s40271-024-00700-y 38878237 PMC11343906

[B20] Pasquini G. Stocking G. Kukuchi E. Pula I. Yam E. (2026). Where do Americans get health information, and what do they trust? Available online at: https://www.pewresearch.org/science/2026/04/07/where-do-americans-get-health-information-and-what-do-they-trust/ (Accessed June 15, 2026).

[B23] Roney C. Egelhofer J. L. Lecheler S. (2026). The “bread and butter” of science: understanding how scientists communicate uncertainties. Humanit Soc. Sci. Commun. 13, 666. 10.1057/s41599-026-07026-0

[B24] Sachsman D. B. Simon J. Valenti J. M. (2008). Environment reporters and U.S. journalists: a comparative analysis. Appl. Environ. Educ. and Commun. 7, 1–19. 10.1080/15330150802194862

[B26] Scutari M. (2025). How Press Forward is Reshaping Local News. Inside Philanthropy. Available online at: https://www.insidephilanthropy.com/home/how-press-forward-is-reshaping-the-nonprofit-local-news-ecosystem (Accessed June 15, 2026).

[B22] The Publication Plan (2025). Why aren’t more journals publishing plain language summaries? Available online at: https://thepublicationplan.com/2025/05/08/why-arent-more-journals-publishing-plain-language-summaries/ (Accessed June 15, 2026).

[B27] Tufte E. R. (2018). The Visual Display of Quantitative Information. Second edition. Cheshire, Conn: Graphics Press.

